# An Integrative Approach to Study Bacterial Enzymatic Degradation of Toxic Dyes

**DOI:** 10.3389/fmicb.2021.802544

**Published:** 2022-01-28

**Authors:** Arti Mishra, Simran Takkar, Naveen Chandra Joshi, Smriti Shukla, Kartikeya Shukla, Anamika Singh, Anusha Manikonda, Ajit Varma

**Affiliations:** ^1^Amity Institute of Microbial Technology, Amity University, Noida, India; ^2^Amity Institute of Environmental Toxicology, Safety and Management, Amity University, Noida, India; ^3^Amity Institute of Environmental Sciences, Amity University, Noida, India; ^4^Department of Botany, Maitreyi College, University of Delhi, New Delhi, India; ^5^Prathista Industries Limited, Hyderabad, India

**Keywords:** toxic dyes, dye degradation, azoreductase, *in silico* studies, molecular docking

## Abstract

Synthetic dyes pose a large threat to the environment and consequently to human health. Various dyes are used in textile, cosmetics, and pharmaceutical industries, and are released into the environment without any treatment, thus adversely affecting both the environment and neighboring human populations. Several existing physical and chemical methods for dye degradation are effective but have many drawbacks. Biological methods over the years have gained importance in the decolorization and degradation of dye and have also overcome the disadvantages of physiochemical methods. Furthermore, biological methods are eco-friendly and lead to complete decolorization. The mechanism of decolorization and degradation by several bacterial enzymes are discussed in detail. For the identification of ecologically sustainable strains and their application at the field level, we have focused on bioaugmentation aspects. Furthermore, *in silico* studies such as molecular docking of bacterial enzymes with dyes can give a new insight into biological studies and provide an easy way to understand the interaction at the molecular level. This review mainly focuses on an integrative approach and its importance for the effective treatment and decolorization of dyes.

## Introduction

**FIGURE 1:**
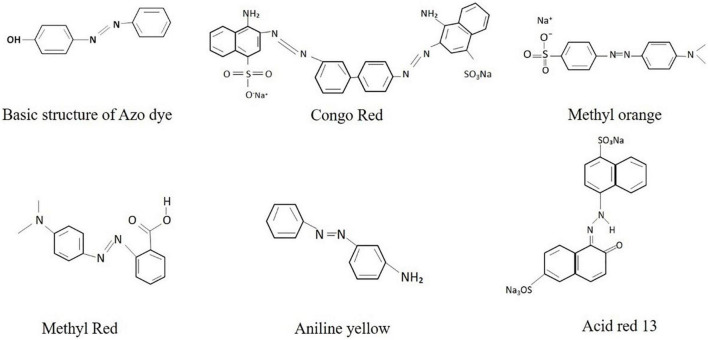
Basic structure of different azo dyes.

For centuries, dyes have been widely used and considered for their coloring property in different industries. There are 700 different available dyes with an estimated 5 tons of yearly production worldwide, out of which two-thirds of the production is dominated by textile industries (Hossen et al., 2019). Dyes containing effluent, discharged directly without any treatment into the nearby water bodies, lead to hazardous effects on the environment (Banat et al., 1996). The major environmental concerns include the biomagnification of toxic dyes at different trophic levels in the food chain and increased value of biological oxygen demand (BOD) as well as chemical oxygen demand (COD), thus disturbing the aquatic life (Lellis et al., 2019). The bioaccumulation of hazardous dyes elevates the teratogenicity, carcinogenicity, and mutagenicity in humans (Wang et al., 2017; Lellis et al., 2019). Earlier in 1994, it was estimated that approximately 1 million tons of dyes were produced worldwide; out of them, 50% were reported as azo dyes (Ollgaad et al., 1999; Adedayo et al., 2004). The azo dyes are synthesized industrially and contribute >50% of the production annually in industrial effluents (Brüschweiler and Merlot, 2017). These dyes are used in various industries such as pharmaceutical, textile, printing, food, and cosmetic industries. Textile effluents released by these dyes in the environment contain high BOD (biological oxygen demand) (80–6,000 mg/L) and COD (chemical oxygen demand) (150–12,000 mg/L), high color (Pt-Co units 50–2,500), alkaline pH (7–10), and high salt concentrations (Suresh, 2014). The chemical structure of azo dyes contains a double bond that is nitrogen–nitrogen (N = N) (Figure 1). Azo dyes are of different types according to their structure. Mono azo dyes have the simplest structure containing one double bond of nitrogen, whereas diazo and triazo dyes contain two and three double bonds of nitrogen, respectively. These dyes are mostly connected to rings like benzene, naphthalene, and some aliphatic rings like heterocyclic. The attachment of azo dyes with rings is crucial because it provides shade and colors to the dye with varying intensities. The azo dyes, when discharged into the ecosystem, get converted into toxic, carcinogenic, and mutagenic aromatic amines that have harmful effects on humans and aquatic life forms. The investigations of biodegradation of azo dyes and their derivative metabolites are of ecological intrigue due to their headstrong cancer-causing nature, mutagenicity, and poisonous impacts. In 2010, Japan reported 26 types of primary aromatic amine production at different concentrations from azo dyes used in textile industries with mutagenic or carcinogenic properties (Kawakami et al., 2010). The issue of visual contamination is more or less understood when dye mixes are converted into their intermediates; however, a more prominent and increasingly malicious issue might be made. More than a thousand dyes are present in the market, out of which 500 or more contain toxic aromatic amines in their chemical structure (Chen, 2006). A survey conducted in 1996 showed that 90% out of 4,000 dyes have LD_50_ values greater than 2,000 mg/kg, with the highest toxicity in azo dyes (Chen, 2006). Moreover, a recent survey conducted by Jiang and co-workers in 2020 showed the assessment of azo dyes and their intermediates in the zebrafish embryos, and to their surprise, the concentration of these dyes in aquatic life is double that of human exposure (Jiang et al., 2020). These observations and data clearly state that the azo dyes are the most dominant and oldest dyes produced, thus requiring immediate action against the unregulated release of the azo dyes.

Through laboratory studies, several researchers have made efforts to deduce the consequences and ecological fate of the azo dyes. Physical and chemical methods are used for removing the dye, such as coagulation-flocculation, adsorption, oxidation, and electrochemical methods (Anjaneyulu et al., 2005; Ajaz et al., 2018; Baena-Baldiris et al., 2020), but have certain disadvantages as mentioned below in Table 1. On the other hand, microorganisms are favored more for the decolorization process because of their unique metabolic pathways and protein frameworks that help them mineralize and decolorize the dye completely under specific ecological conditions. The microorganisms that decolorize various azo dyes are fungi (Bankole et al., 2018; Jasińska et al., 2019; Usha et al., 2020), algae (Mahalakshmi et al., 2015; Raymond and Kadiri, 2017; Ishchi and Sibi, 2020), yeast (Yu and Wen, 2005; Mahmoud, 2014), and bacteria (Hossen et al., 2019). Microbial degradation of azo dye occurs under aerobic, anaerobic, or microaerophilic conditions. However, anaerobic processes lead to the formation of aromatic amines, which are toxic than their parent dye and highly carcinogenic to humans (Sathishkumar et al., 2017). Out of all these organisms, bacteria have gained much attention due to their high degree of decolorization compared with other microbes (Saratale et al., 2011). The initial step in bacterial degradation and decolorization is the reduction of the azo bonds by enzymatic cleavage, which leads to the formation of aromatic amines (Pandey et al., 2007; Singh et al., 2017). Different enzymes such as laccase, lignin peroxidase, and azoreductase are involved in the dye degradation process by degrading the intermediates formed in the decolorization, following different pathways’ biodegradation (Kumar et al., 2018; Falade et al., 2019; Ameenudeen et al., 2021).

**TABLE 1:** Advantages and disadvantages of the physical and chemical techniques.

Physical/chemical method	Advantage	Disadvantage	Explanation
Fenton reagent	Effective decolorization of both soluble and insoluble dyes	Sludge generation	Oxidation reaction using mainly H_2_O_2_-Fe(II)
Ozonation	Applied in gaseous state: no alteration of volume	Short half-life (20 min)	Oxidation reaction using ozone gas
Photochemical	No sludge production	Formation of by-products	Oxidation reaction using mainly H_2_O_2_-UV
NaOCl	Initiates and accelerates azo-bond cleavage	Release of aromatic amine	Oxidation reaction using Cl^+^ to attack the amino group
Electrochemical destruction	Breakdown compounds are non-hazardous	High cost of electricity	Oxidation reaction using electricity
Activated carbon	Good removal of a wide variety of dyes	Very expensive	Dye removal by adsorption
Membrane filtration	Removes all dye types	Concentrated sludge production	Physical separation ion exchange
Ion exchange	Regeneration: no adsorbent loss	Not effective for all dyes	Difference in interaction of reactants with ion exchange resin
Irradiation	Effective oxidation at laboratory scale	Requires a lot of dissolved O_2_	Reaction is controlled by the radiation dose and the availability of oxygen in solution
Electrokinetic coagulation	Electrokinetic Coagulation	Electrokinetic coagulation	Addition of ferrous sulfate and ferric chloride

This review mainly focuses on an integrative approach for the treatment and decolorization of dyes. The mechanism of decolorization and degradation by several bacterial enzymes and bacteria are discussed in detail along with different remediation approaches. The application and hazardous effects of dyes at cellular and chronical level are also mentioned. Furthermore, we have discussed *in silico* studies with dyes to give new insight to understand the interaction at the molecular level.

## Ecotoxicity Based on the Chemical Characterization of Azo Dye

**FIGURE 2:**
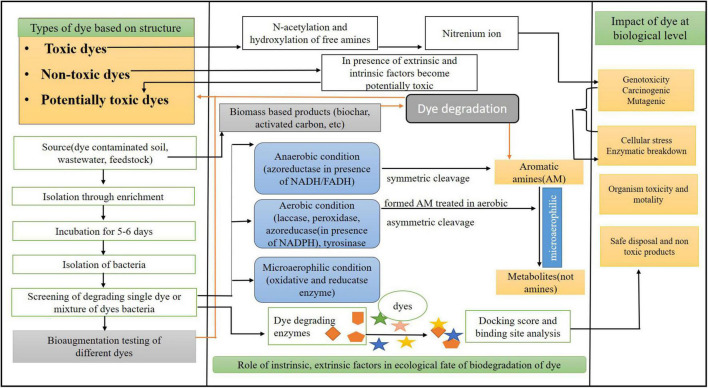
A schematic diagram showing an integrated approach of interaction of dye with different approaches of degradation and intrinsic/extrinsic factors.

Based on the laboratory testing, azo dyes are divided into two categories: toxic and non-toxic dyes. Toxic dyes induce the free and N-acetylated amino groups in dyes that form the RNA/DNA nitrenium bindings, thus making the dye genotoxic, as shown in Figure 2. These dyes are mostly hydrophobic (taken up via bacterial cell and reduced in the cell and causes cellular and ecological impacts), carcinogenic, mutagenic, and lipophilic. Non-toxic azo dyes are non-carcinogenic due to the presence of alkyl, arylamines, and the addition of carboxyl and sulfo groups. When non-toxic azo dyes interact with biotic and abiotic factors, they become potentially toxic. The current classification of the unregulated non-toxic potential azo dyes has been ignored as the dyes associated with toxicity have attained more attention from the researchers. These findings suggest that the interaction between the environmental factors and the chemical structure responsible for the toxicological effects can form the basis for differentiating toxic and non-toxic azo dyes. The metabolites produced cause the formation of cytogenic, genotoxic, and carcinogenic compounds, which inhibit the growth of the microbes (Chung et al., 1997) and can also lead to changes in the DNA synthesis (Joachim and Decad, 1984). Most of the azo compounds are xenobiotic, and the one xenobiotic combination with the natural azo compound was reported that is 4–4’ dihydroxy azobenzene (Pandey et al., 2007).

Various environmental factors like biotic factors, abiotic factors, and chemical properties under natural conditions play a significant role in the ecotoxicity of azo dye. However, the assumptions on the ecotoxicological impact of azo dyes under natural conditions have not been studied and proposed yet. Currently, it is impossible to assess each dye’s ecotoxicity with azo dyes’ broad and diverse structure as it also adds to the cost.

## Processes Involved in the Remediation of Dye

### Physiochemical Reduction Methods

Physical methods are essential methods that include the removal of sulfur and disperse dyes based on the flocculation and coagulation of dyes. In contrast, it displays less flocculation and coagulation for dyes like direct, reactive, vat, and acid dyes. Techniques like flocculation, coagulation, membrane filtration, precipitation, adsorption, irradiation, Fenton oxidation, and irradiation are included in physiochemical methods. Ironically, these methods have drawbacks as they require more chemicals and energy, and are not able to completely decolorize the dye; it is also reported that the usage of these methods generate excessive sludge and secondary pollutants (Khan et al., 2014; Tiar et al., 2018) and cause secondary land pollution (Roy et al., 2018). Moreover, these methods do not solve the problem completely but instead transfer the dye from one phase to another. Mostly this treatment method is reported as not a good option for the degradation and decolorization of dye because of their incomplete conversion to CO_2_ (Hassan et al., 2013). It was reported that the use of methods like ozonation, electrocoagulation, adsorption, activated carbon, froth flotation, reverse osmosis, and ion exchange are not much efficient, rather they are very costly, and produce water and compounds that are difficult to get rid of Mahmood et al. (2015). Therefore, this states that physiochemical methods have been applied for a longer time but have increased ecotoxicity. Various advantages and disadvantages of different physical and chemical techniques have been mentioned in Table 1.

### Biomass-Derived Adsorbents of Toxic Dye

The ability of the biomass feedstock in the production of carbonaceous adsorbents (CAs) and their application in dye removal have gained importance in recent years because of their high dye binding capacity. The ideal feedstocks for the CAs are waste materials because of their enrichment in carbon. Various plant and crop residues (such as wheat straw, switchgrass, weeds, bamboo, and jute fiber), fruit and tree residues (such as wood waste, sawdust, orange peel, coconut shell, lignin, coconut flower, pine fruit shell, and almond shell), animal finery waste, municipal organic solid waste, and fresh marine biomasses are investigated for CA. Various CAs produced from feedstock studied in dye removal are biochar, activated carbon, nanostructured carbon, etc (Figure 2).

Biochar prepared from Ashe juniper (Choi et al., 2019), pine cone (Dawood et al., 2017), wheat straw (Li et al., 2016), wood waste (Kelm et al., 2019), and switchgrass (Mahmoud et al., 2016) have been investigated for the removal of dyes from dye effluents. Biochar prepared from the hydrothermal method (Islam et al., 2017) is also studied to remove the dye. The most popular adsorbent is activated carbon (AC) with low to high surface area. The pore structure of the adsorbents and surface area of AC affect the dye-adsorbing properties (Wu et al., 2017). In 2016, AC produced from almond shells showed the second-highest reactive dye to the Reactive Red 2 and Reactive 413 Yellow 145A with high dye binding of 1,639.9 and 1,397.4 mg/g, respectively (Thitame and Shukla, 2016). Commercial AC was investigated as adsorbents for reactive, acidic, and basic dyes (Machado et al., 2011). Various nanostructured carbons such as carbon nanotube, graphene, and graphite are investigated mainly to remove toxic stains as an adsorbent. Graphene oxide (Mao et al., 2020), graphene (Liu et al., 2016; Elsagh et al., 2017), and functionalized MWCNT (Karimifard and Alavi, 2016) have been studied for reactive dye removal.

### Nanobiotechnology and Innovative Approaches

Apart from microbial decolorization, various other approaches are used by researchers in their laboratory for the degradation of toxic compounds that are harmful to humans and the environment. One such approach is the use of biopolymers. Biopolymers are biodegradable, non-toxic, and renewable. Chitosan is a type of biopolymer that can be easily modified and can be used in degradation. Recent research on the preparation of chitosan for the removal of methyl orange from aqueous solution was seen (Yuvaraja et al., 2019); modified chitosan has helped degrade tetracycline (Farhadian et al., 2019). The use of chitosan gave the leading edge by forming silver nanoparticles, which degraded the toxic phenol (Ranjani et al., 2019). Nanotechnology also is a diverse field that can be used in the bioremediation process. Nanoparticles can be used in the degradation process. Many researchers have justified this by performing the degradation by the formation of nanoparticles. The formation of copper nanoparticles was shown to decolorize the Safranin, Malachite green, and Carbol fuchsine dyes and showed positive results (Dlamini et al., 2019). The photocatalysis method for degrading dye has shown its advantage over the oxidative treatment processes and is widely used by researchers to degrade dye.

Removal and decolorization of brilliant red X-3B have been successfully done by combining photocatalytic and biocatalysis (Zhang et al., 2017). It was reported that nanosheets complex with silver cations led to the 90% decolorization of methyl blue via photocatalytic activity (Kadam et al., 2019). Using nanocomposites as photocatalysts also have shown positive results in decolorizing titan yellow and methyl orange dyes (Kumar et al., 2020). Coupling hydrogen peroxide H_2_O_2_ with the iron–carbon micro-electrolysis is a new eco-friendly method and has shown positive results in decolorizing Direct Blue 15 dye (Yang et al., 2018). A very different approach of coupling inductively coupled plasma mass spectrometry (ICP-MS) and time-of-flight mass spectrometry (Q-TOF-MS) was performed by researchers to decolorize the Reactive Orange 107 dye under anaerobic and aerobic conditions (Frindt et al., 2017). Metal-organic frameworks are nanoporous structures that have recently shown better adsorption of dyes under different physiochemical methods (Ayati et al., 2016). Therefore, besides using microorganisms for bioremediation processes, nanotechnology methods can also remove the dyes from the environment and reduce their toxic effects.

### Biological Reduction Methods

The biological remedy is an attractive opportunity for the development of an efficient treatment process for textile dyes. Biological treatment methods lead to the complete decolorization and mineralization of dye and are eco-friendly methods that can be done at a low cost (Pandey et al., 2007). Over the last two decades, this method has gained enormous popularity (Anjaneyulu et al., 2005). In biological processes, microbes can acclimatize themselves to toxic wastes, which can help them develop resistance to toxic traces. This method can also change organic pollutants into various forms like water, CO_2_, and inorganic salts (Ewida et al., 2019). This process has proven to be effective for decolorization and degradation, and the mechanisms by which they degrade have been explored (Stolz, 2001; Rai et al., 2005; Van der Zee and Villaverde, 2005; Ghosh et al., 2017; Mani et al., 2019) either by isolation of organisms as pure cultures or the multispecies culture. The diversity, versatile nature, and metabolic pathways of the organisms play a vital role in degrading different dyes. In general, microbial decoloration can occur via two fundamental mechanisms: biosorption and enzymatic degradation, or a mixture of both processes. Microorganisms like bacteria, yeasts, fungi, and algae can completely decolorize and mineralize the dyes under specific conditions. The major advantages of this method include eco-friendly, less costly, no sludge, and complete decolorization of the dye. The degradation of dyes by biological methods is mostly detected with analytical techniques such as UV–vis spectroscopy, Fourier transform infrared radiation spectroscopy (FTIR), high-performance liquid chromatography (HPLC), and gas chromatography–mass spectrometry (GC-MS).

#### Bacterial Reduction

Bacterial decolorization of synthetic dyes has become popular because they are eco-friendly, utilize less water than the other physical and chemical methods, and decolorize different dyes (Tiar et al., 2018). Degradation by bacteria allows its metabolic pathways to use xenobiotic compounds as a substrate. It was seen and reported that bacterial decolorization processes either involve the use of pure bacterial culture (Lin et al., 2010; Haq and Raj, 2018; Patil et al., 2018; Chittal et al., 2019) or the multispecies consortium (Das and Mishra, 2017; Cao et al., 2019; Kumari and Rajoriya, 2019). Bacteria used to dispose of dyes and decolorize a huge spectrum of dyes that have been isolated and characterized are listed in Table 2. Bacterial cultures are favored over other microorganisms, mainly fungi, in the case of anthraquinone dye (Balasubramanian et al., 2011; Li et al., 2019) because bacteria can be grown fast, can survive in harsh conditions, and are easy to manipulate. A recent study identified azoreductase and naphthalene-degrading genes in bacteria found near seashore that decolorized naphthalene-based dyes (Zhuang et al., 2020). Therefore, bacteria can reduce the ecotoxicity of the azo dyes and decolorize the vast array of dyes like amaranth, naphthalene, and acidic and anthraquinone dyes. The biodegradation of azo dyes may occur either aerobically, anaerobically, or by a combination of both discussed in the following section.

**TABLE 2:** Decolorization of dyes by bacteria.

Dye	Bacteria	Conditions Temp (°C), pH, agitation	Decolorization	Enzymes involved	Time	References
Sumifex Tourqi blue	*Alishewanella* sp. CBL-2	37, pH 7, NA	83%	Reductive (azoreductase)	6 days	Ajaz et al., 2018
Methyl Red	*Pseudomonas nitroreducens Vibrio logei*	30–35, 6, NA	100% decolorization by *Vibrio logei*, 60% by *Pseudomonas nitroreducens*	NA	15 h	Adedayo et al., 2004
Novacron Super Black G	*Bacillus* sp.*Alcaligenes faecalis* kaz26	37, 8, static, shaking	90% by both	NA	96 h	Hossen et al., 2019
Reactive Orange 16	*Neocardia* sp.	35, 8, NA	85.6%	NA	24 h	Chittal et al., 2019
Crystal Violet	*Enterobacter* sp.	35, 6.5, shaking	100%	NA	72 h	Roy et al., 2018
Orange W3R, red FNR, yellow FN2R, blue FNR, and navy WB	*Micrococcus luteus*, *Listeria denitrificans*, *Nocardia atlantica*	NA, NA, shaking	Up to 80% decolorization of all the dyes, but *N. atlantica* completely decolorized blue FNR, red FNR	NA	7 days	Hassan et al., 2013
Dark Red 2B	*Klebsiella* sp. *Staphylococcus* sp.	37, 6, static 37, 7, shaking	98.83% 98.72%	NA	5 days	Desai, 2017
Reactive Red 195	Bacterial consortium AR1	40, 8, microaerophilic	100%	Reductive (azoreductase)	14 h	Khan et al., 2014
Azure B	*S. liquefaciens*	25–30, 5–7, shaking	Up to 96%	Oxidative (LiP)	48 h	Haq and Raj, 2018
Xylidine Ponceau 2R	*S. marisflavi* EP1	35, 7, anaerobic 2% NaCl	Up to 100%	NA	17 h	Xu et al., 2016
Reactive Black 5	*P. entomophila* BS1	37, 5–9, static	93%	Reductive (azoreductase)	120 h	Khan and Malik, 2016
Reactive Green-19	Consortium M1C and M2C	30–35, 8, static	More than 97%	NA	24 h	Das and Mishra, 2017
Acid red 337	*Bacillus megaterium*	30, 7, NA	91%	NA	24 h	Ewida et al., 2019
Orange MR	*Micrococcus* sp.	35, 6, NA	Up to 95%	NA	48 h	Rajee and Patterson, 2011
Synazol Red 6HBN	*Alcaligenes aquatilis* 3c	37, 7, static	86%	NA	5 days	Ajaz et al., 2019a
Reactive Violet	*Paracoccus* sp. GSM2	25–40, 6–9, static, aerobic	100% in static 16% in shaking	NA	16 h	Bheemaraddi et al., 2014
Methyl Red	*Lysinibacillus fusiformis* W1B6	30, 6–8, static, aerobic	97% in static, 96% in shaking	Azoreductase, laccase, lignin peroxidase (LiP)	2 h	Sari and Simaran, 2019
Methyl Red	*Sphingomonas paucimobilis*	30, 9, shaking	Up to 100%	NA	10 h	Ayed et al., 2011
Acid Black 24	*Bacillus pseudomycoides*	37, 7, static, shaking	Up to 95% in static, 20% in shaking	Oxidoreductase LiP, laccase azoreductase	25 h	Kumar et al., 2019
Direct Blue 151 Direct Red 31	Bacteria Consortium	36–45, 8.5–9.5, NA	97.57% 95.25%	NA	5 days	Lalnunhlimi and Krishnaswamy, 2015
Congo Red	*Enterobacter* sp. SXCR	34, 7, static, shaking	57% in static, 6% in shaking	Reductive (azoreductase)	48 h	Prasad and Aikat, 2013
Reactive Red 2	*Pseudomonas* sp. SUK1	30, 6.2–7.5, static	52%	Oxidoreductase (azoreductase, LiP)	24 h	Kalyani et al., 2008
Congo Red	*Bacillus* sp. Consortium	37, 7, static	99%	NA	14 h	Kumari and Rajoriya, 2019
Remazol Black B	*Pseudomonas aeruginosa* KY284155	NA, 9, static, shaking	74% in static 4% in shaking	Reductive (azoreductase)	48 h	Hashem et al., 2018
Remazol Brilliant Blue R	*Staphylococcus* sp. K2204	37, 7, static	100%	Oxidative (laccase, LiP)	12 h	Velayutham et al., 2018
Malachite green	*Strain* T-5-2	27, 6.5, NA	NA	MG reductase	49 days	Kobayashi et al., 2017
Reactive Blue 4	*Staphylococcus hominis* subsp. *hominis* DSM 20328	37, 7, static	100%	Reductive (azoreductase, NADH-DCIP)	25 h	Parmar and Shukla, 2018
Direct Blue 2B	*Bacterial consortium* YHK	37, 6–8, static, shaking	Up to 85% in static, 60% in shaking	Oxidoreductase (azoreductase, laccase)	48 h	Cao et al., 2019
Reactive Blue 160	*Bacillus firmus*	NA, NA, NA	100%	NA	NA	Barathi et al., 2020
Methyl Red	*Bacillus circulans* NPP1	35, 7.5, static	98%	Oxidoreductase (azoreductase, lignin peroxidase laccase, and tyrosinase)	4 h	Patil et al., 2018

#### Aerobic, Anaerobic, and Microaerophilic Treatment in Dye Degradation

Aerobic biological treatment is not much efficient for decolorizing the dye effluents mainly because of two reasons: (1) dyes are mostly stable to biological oxidation and so can be considered as oxidative stable, and (2) this method mostly leads to the inefficient adsorption of dyes on the activated sludge (Keharia and Madamwar, 2003). It was seen that bacteria in aerobic treatment mostly show their activity for sulfonated azo dyes in which aerobic organisms are required to be specially adapted but are sometimes difficult to isolate as reported in Franciscon et al. (2012), whereas in an anaerobic environment, sulfate-reducing bacteria form hydrogen sulfide, which has resulted in the reduction azo dyes such as Reactive Red 120 (Stolz, 2001). Azoreductase enzymes help in the decolorization of dye in anaerobic conditions; similarly, mono and di-oxygenase enzymes are produced in aerobic conditions that cleave the azo bonds. Moreover, in aerobic conditions, oxidative enzymes also work well as reported by Sarayu and Sandhya (2010). It was shown that the metabolites formed in the aerobic environment of para red and sudan I dyes led to oxidative bursts, mutations, and chromosomal damage in human cells. From the aforementioned observation, we can propose that both oxidative and reductive conditions can lead to an increased level of toxicity. Zimmermann et al. (1984) gave the earliest example of azo reductases working under aerobic conditions in *Pseudomonas* sp. KF46 (Sari and Simaran, 2019). It has been observed that aerobic bacteria mostly show high specificity to most of the dye structures and require a longer period of acclimatization in the presence of azo compounds to induce the expression of azoreductase (Cui et al., 2014). The bacteria *Klebsiella* sp. and *Staphylococcus* sp. has decolorized up to 90% dye under aerobic condition (Desai, 2017). Almost complete decolorization was reported by *Sphingomonas paucimobilis*, unidentified bacterium KMK, and *Enterobacter* sp. CV-S1 (Kodam et al., 2005; Ayed et al., 2011; Roy et al., 2018). However, Giordano in 1992 performed the aerobic microbial degradation of Acid Red 151 (AR151), in which azo bonds were cleaved and yielded aromatic amines along with some carcinogenic metabolites, which can be identified by GC-MS analysis. However, in some cases, aerobic treatments have not shown positive results in cases as mentioned in Table 2 (Kalyani et al., 2008; Prasad and Aikat, 2013). As compared with the aerobic environment, anaerobic degradation follows the two mechanisms for the decolorization of dyes: (1) first, it involves the direct transfer of electrons to dyes as terminal acceptors with the help of enzymes (Ryan et al., 2010; De Souza et al., 2018); (2) the second step follows the reduction of dye with the help of the bacteria catabolism (e.g., reduction of the bond via decreased inorganic compounds, inclusive of Fe^2+^ or H_2_S, which can be shaped as the stop product of sure anaerobic bacterial metabolic reactions). Anaerobic degradation of azo dyes with the help of bacteria is an economical and effective method for decolorization because most of the reactions occur at neutral pH (Gopinath et al., 2009). Furthermore, this type of degradation is non-precise, while low molecular weight redox mediators are also present. Decolorization under static conditions comes under anaerobic treatment only. In the static condition, oxygen depletion is readily achieved without any problem, so this condition allows the obligate and facultatively anaerobic organisms for the cleavage and reduction of azo dyes. Mostly degradation in static conditions occurs at neutral pH (Kalyani et al., 2008; Ajaz et al., 2019a). Decolorization under anaerobic conditions is usually not much specific; anaerobic or static conditions for the removal of dye is more efficient than aerobic decolorization (Xu et al., 2007; Joe et al., 2008; Tiar et al., 2018). In both anaerobic and aerobic conditions, glucose can be used as a co-substrate, which helps in the faster reduction, but sometimes carbon and glucose sources may lead to incomplete degradation and release the aromatic amines, which are hazardous and toxic (Stolz, 2001). One of the advantages of this method is that the depletion of oxygen can be achieved without any problem in static cultures, which allows facultative anaerobic, anaerobic, and aerobic microorganisms to reduce the azo dyes. Mostly azo dyes are degraded; however, the toxic aromatic amines formed from these azo dyes cannot be fully degraded in anaerobic conditions, so as a result, there is the accumulation of poisonous substances, mostly aromatic amines. Then these aromatic amines are degraded in aerobic conditions, so the combined aerobic–anaerobic method is better for completely degrading the dye (Figure 2). Melgazo reported the decolorization of Disperse blue 79 via anaerobic–aerobic bioreactors (Gao et al., 2017). An example of anaerobic–aerobic method was also seen by colorant Disperse blue 79 (DB79), which was converted into aromatic amines in the anaerobic phase by microbes and then the further mineralization of aromatic amines occurs in the aerobic phase, which mostly eliminates the toxicity of the dye. The anaerobic decolorization method is not able to decolorize and mineralize the dye completely and its toxic products. Moreover, the aerobic method for decolorizing the dyes is not favored to remove dye from wastewater plants. Due to the drawbacks of the anaerobic and aerobic treatment, researchers mostly prefer the microaerophilic method, which is considered the best method (Gao et al., 2017; Ajaz et al., 2019b). The sequential anaerobic–aerobic treatment is the easiest and most feasible method under the biological treatment for the dye removal as these methods have shown positive results in Fast Acid Red GR and Dark Red 2B (Xu et al., 2007; Desai, 2017). A very innovative approach of using the bioreactor in which rice husks were used as organic material to support the growth of organisms has been reported in which the anaerobic–aerobic bioreactor showed better effect than aerobic bioreactor alone (Pereira et al., 2019). The positive results of the sequential aerobic–anaerobic and anaerobic–aerobic process showed that bacterial consortium of fungi and bacteria could decolorize the dye within 30 h (Lade et al., 2015). However, under aerobic conditions, a very less amount of dye was removed by the consortium. A similar observation was seen in which bacterial consortium under microaerophilic conditions could decolorize the four dyes within 12–30 h (Lade et al., 2015). The simultaneous system utilizes the anaerobic zones in the anaerobic phases found in granular sludge, biofilms, or immobilized biomass. The fluctuations in the azo dyes’ fate lead to disastrous impacts on the ecosystem and humans. However, the current toxicity of assessment of azo dyes has led to the research on the hidden hazardous impacts, but the aforementioned facts have emphasized the need for a more ecological toxic assessment for better management of azo dyes.

### Bioaugmentation of Dye Toxicity

The microorganisms could also be indigenous to a contaminated area or isolated from elsewhere and delivered to the contaminated site by living organisms through reactions that happen as a neighborhood of their metabolic processes. The biodegradation of a compound is usually a result of the actions of multiple organisms. When microorganisms are imported to a contaminated site to reinforce degradation, the method is named “bio-augmentation.” Recently, bacterial and fungal microbial communities isolated from secondary sludge CETP161 Vatva, Ahmedabad, showed a decolorization rate of 972 ± 1.21 mg dyes kg soil^–1^ day^–1^ after 15 days from soil (Patel et al., 2020). The microorganisms with the genetic capacity to rework compounds of interest must be present in contaminant metabolism during bioremediation. In some instances, the addition of organisms acclimated to specific contaminants or bioaugmentation may decrease the duration of lag phases. The ability to effectively bio-augment the bioremediation system may be a function of the method used. Biological reduction by isolating bacteria mentioned in the previous section and bioaugmentation for bioremediation are interconnected, as shown in Figure 2. Bioremediation is an alternative that offers the possibilities to destroy, or render harmless, various contaminants using natural biological activity (Gupta and Mahapatra, 2003; Singh R. et al., 2014; Tiwari et al., 2015).

The effectiveness of individual isolates can often be enhanced by co-culture with other highly efficient dye-decolorizing strains (Chen et al., 2006; Khalid et al., 2010). Here, it is speculated that the combined enzyme systems of the mixed bacterial culture are more straightforward than the enzymes from the individual isolates, each of which can have different substrate kinetics and efficiency at different dye concentrations. Cooperation within microbial communities also can occur through the exchange of growth cofactors and the removal of toxic metabolites. Although many microorganisms can degrade azo dyes (Chen et al., 2006; Hao et al., 2007; Khalid et al., 2010), relatively few microbial species and strains have emerged as candidates for use in bioaugmentation (Chen et al., 2006; Khalid et al., 2010). Thus, before individual isolates can be recommended, comprehensive research is required to understand the role of individual microorganisms and their interactions with the other microflora (Pinheiro et al., 2004).

## Impact of Extrinsic and Intrinsic Factors for the Removal of Dyes

### Extrinsic Factors

Microbial decolorization and degradation are influenced by temperature because different microorganisms show their best activity at a defined variety of temperatures. As the temperature increases, the process of decolorization increases up to a limit, whereas after reaching the optimum limit, reduction in decolorization process is observed. Mostly, the narrow temperature range for the decolorization of dyes is reported, consistent with bacterial growth between 20 and 37°C (Cui et al., 2016; Haq and Raj, 2018; Velayutham et al., 2018; Hossen et al., 2019). After this range, the increase in temperature decreases the decolorization process, but certain bacteria have been shown to decolorize between 40 and 50°C (Mahbub et al., 2012; Bhattacharya et al., 2017; Patil et al., 2018). The temperature increased to and after 55°C may result in the loss of the growth of cells or the deactivation of enzymes mainly responsible for the decolorization (Chang et al., 2001; Parmar and Shukla, 2018). Therefore, the 30–45°C temperature range is an optimum temperature for the organisms to remove the maximal dye. At present, printing and dyeing of wastewater as the remedy plants usually do not work in the cold season because most bacteria’s growth is reduced at the low temperature, thus influencing the degradation and decolorization of dye. However, the isolation of bacterial strains that can work at higher temperatures will be essential in future research. In addition, some studies have shown that the activation energy required for the microbial decolorization of azo dyes has been reported (Santos et al., 2007), whereas the narrow temperature degrees are chosen as the best temperatures for the decolorization of dyes by the pure cultures or the consortium of microorganisms inhabiting active sludge.

The pH also plays an essential factor in the decolorization process. It is observed that the decolorization mainly occurs at neutral pH and a slightly alkaline pH. Mostly it has been seen that the optimum pH for the decolorization is between 6.0 and 8.0 (Khan et al., 2014; Singh R. P. et al., 2014; Vikrant et al., 2019). Meanwhile, the removal efficiency mostly decreases under high acidic or alkaline conditions (Cui et al., 2014; Kobayashi et al., 2017; Chittal et al., 2019). In 2018, a study reported that Remazole dye was able to decolorize at pH 9 effectively and showed less or no result at pH 5–6 (Hashem et al., 2018). The rate of decolorization at neutral pH 7 has been observed in various dyes (Table 2). The removal efficiency is affected by pH in two ways: (1) it changes the molecules of dye in an aqueous solution thus affecting the dye absorption process, and (2) it might also affect the permeation efficiency of the cellular membrane, which acts as a restricting step in the bacterial decolorization and degradation. Aromatic amines formed by reducing azo bonds can lead to a change in the pH because these formed amines are more basic than the parent azo dye. A buffer is typically introduced to modify the pH value for the duration of the biological process in wastewater plants that enhance the rate of removal and the activity of bacteria. Optimizing the pH of bacteria is necessary to improve their decolorization efficiency. If the concentration of the dye is shallow, the enzymes secreted from the degradative dye find it difficult to identify the dye, and when the concentration of dye is too high, it blocks the active sites of the bacteria and becomes toxic to the bacteria (Jadhav et al., 2008). However, in the initial stages of dye concentration, there is an increased rate of decolorization, which gradually decreases when the concentration of dye is increased to high concentration (Ghodake et al., 2011; Rajee and Patterson, 2011; Cui et al., 2014; Bhattacharya et al., 2017; Patil et al., 2018; Ewida et al., 2019). Dyeing wastewater has variable quality of water with the distinction of chromaticity reaching around 4,000 times, which has led the researchers to acclimatize the potential degradative bacteria that may help these bacteria to adapt to different concentrations. With increased concentration, there is a decrease in decolorization, which can be explained as an inverse proportional relationship in most cases between the dye concentration and the activity of bacteria. This decrease in decolorization may happen because of the inadequate biomass and blockage of enzymes due to the toxicity of the dye. Thereby, apart from the chemical structure and the unpredictable fate, the role of these environmental factors has extended the level of research in this particular direction.

### Intrinsic Factors

Proteomics enzymes are involved in the different biochemical reactions that occur in microorganisms, and because of their catalytic property, these can be used in the bioremediation process. Recently, the enzymatic approach has gained the interest of researchers for the decolorization and degradation of dyes in the fabric and textile industry and from wastewater as an alternative strategy to existing physiochemical methods. The bacterial decolorization of dyes occurs by different enzymes like reductive enzymes including azoreductase, nicotinamide adenine dinucleotide-2,6-dichlorophenolindophenol (NADH-DCIP) reductase, magnesium (MG) reductase, and various oxidative enzymes like lignin peroxidase (LiP) and laccase.

#### Azoreductase

**FIGURE 3:**
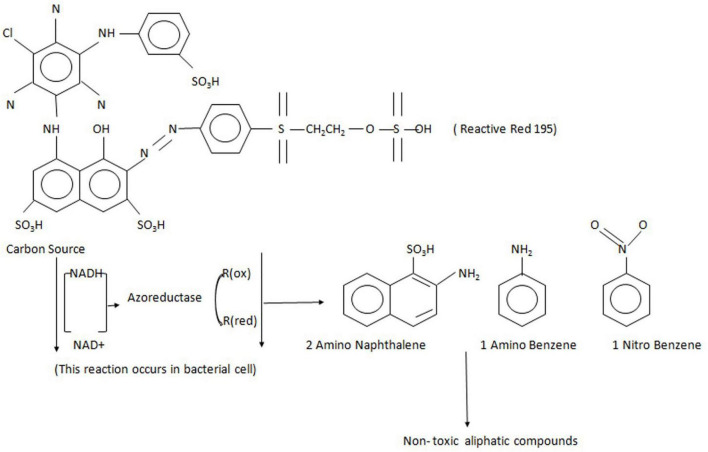
The mechanism action of azo reductase on Reactive Red 195 dye, which forms the aromatic amines and further gets degraded to non-toxic aliphatic compounds.

Azoreductases are the reductive enzymes that catalyze the reductive cleavage of azo bonds to produce aromatic amine products that are mostly colorless. These enzymes are the NAD(P)H:flavin oxidoreductase, which are the flavoproteins. The mechanism of azoreductase is explained in Figure 3 with an example of the degradation of Reactive Red 195 azo dye (Khan et al., 2014). These enzymes are also present in either the extracellular or intracellular area of the bacterial cell membrane and are observed in the intestinal microbiota (Zahran et al., 2019). Many researchers have cloned and characterized azoreductase enzymes from various bacteria and have studied their activity on dye degradation (Qi et al., 2016; Cao et al., 2017; Rathod et al., 2017; Verma et al., 2019). For example, the azoreductase from the *Halomonas* sp. reported its optimum temperature at 20°C (Tian et al., 2018), whereas the azoreductases’ optimum temperatures for *Brevibacillus laterosporus* TISTR1911 and *Pseudomonas* sp. are at 40 and 30°C (Lang et al., 2013; Elfarash et al., 2017). Most of the azoreductase work at the optimum pH of 6–8, but some can work at acidic conditions as reported by azoreductase from *Streptomyces* sp. (Dong et al., 2019). Azoreductases require electron donors like NADH or NADPH, or FADH for the cleavage of an azo bond. The azoreductases have their substrate specificity that mostly depends on the organization of functional groups close to the azo bond. The enzyme azoreductases from bacteria represent a class of enzymes that show similarity with other reductive enzymes. For example, the *Bacillus subtilis* azoreductases AzoR1 and AzoR2, which have around 30% amino acid sequence identities, and *E. coli* AzoR can be used by electrophiles including catechol, 2-methyl hydroquinone (2-MHQ), and diamide (Leelakriangsak et al., 2008).

#### Nicotinamide Adenine Dinucleotide-2,6-Dichlorophenolindophenol and Magnesium Reductase

Nicotinamide adenine dinucleotide-2,6-dichlorophenolindophenol reductases act as the marker enzymes of the bacterial and fungal mixed characteristic oxidase, which helps in detoxifying the xenobiotic compounds. These marker enzymes cause the reduction of the azo bond, and when this enzyme is present, the 2,6-dichlorophenolindophenol (DCIP) takes an electron from the NADH to shape its leuco form. The DCIP enzyme is blue in the oxidized state, but when it is reduced, it becomes colorless. The action of NADH-DCIP reductase has been seen in the *Bacillus* sp. ADR, which was able to decolorize Reactive Orange 16, Red Lb1 dye by *Pseudomonas* sp. SUK1, methyl red by *Brevibacillus laterosporus* (Gomare and Gowindwar, 2009), and Direct Brown MR by *Acinetobacter calcoaceticus*. However, the NADH-DCIP enzyme showed the highest contributing activity for decolorizing the amaranth dye irrespective of the other enzymes present (Ghodake et al., 2011). The decolorization of malachite green by the non-specific reductase is called MG reductase. Through the help of using NADH as an electron donor, these MG reductive enzymes reduce the malachite green into leucomalachite green (Parshetti et al., 2006; Kobayashi et al., 2017; Du et al., 2018).

#### Riboflavin Reductase

Riboflavin reductase is an enzyme which catalyses the reduction of unfastened flavins by NADH, and NADPH. Due to this, flavoprotein and enzyme flavin reductase or NAD(P)H:flavin oxidoreductase is used (Ingelman et al., 1999). This enzyme catalyzes the flavoproteins into the reduced flavins [flavin denine dinucleotide (FAD) or flavin mononucleotide (FMN)] with the help of re-oxidation of nicotinamide adenine dinucleotide (NADH) or nicotinamide adenine dinucleotide phosphate (NADPH), which are in the reduced form. Then these reduced flavins transfer the electrons to the molecules of dye (terminal electron acceptor), which reduces the azo bonds and then gets re-oxidized. The position of riboflavin reductase became the important factor in the decolorization of Mordant Yellow 10 with the help of anaerobic granular sludge (Field and Brady, 2003). In general, flavoproteins capable of reducing the azo dyes differ by the microorganism in which they are observed. For example, FMN prosthetic group, present in both *Proteus vulgaris* and *Staphylococcus faecalis*, helps in catalyzing the azo bond and riboflavin, FMN, or FAD is formed in the same quantity in *S. faecalis*.

#### Laccase

Laccases are copper-containing enzymes that catalyze the few inorganic compounds and the substrates of the oxidation process like phenols, ascorbic acid, arylamines, and anilines. These enzymes are primarily found in bacteria, lichens, plants, fungi, insect cuticles, and metagenomic libraries of the bovine rumen. These are differentiated into two types: (1) blue laccase, which is blue and shows specific EPR spectra; (2) white or yellow-brown laccases, which show unusual EPR spectra. These enzymes are also called phenoloxidase, which helps in catalyzing the oxidation of different fragrances, phenols, and inorganic substances with the associative conversion of water to oxygen. Many researchers have cloned and characterized laccase from various bacteria and have studied and reported their activity on dye degradation (Koschorreck et al., 2008; Neifar et al., 2016; Liu et al., 2017; Kumar et al., 2018; Wang et al., 2019). Bacterial laccases mostly show their activity under the neutral to alkaline pH range and can also work in a wide temperature range (Yang et al., 2017). However, some cloned laccase from fungi has shown its activity at a higher temperature around 60°C (Yang et al., 2020), whereas laccase from bacteria recently has shown its activity up to 90°C (Unuofina et al., 2019). *Paenibacillus glucanolyticus* and *Bacillus amyloliquefaciens* laccase produce non-blue laccases, which have been shown to work in acidic conditions, but the optimum temperature of non-blue laccases was less as compared with present research findings (Chen et al., 2015; Mathews et al., 2016). Recently, a very advanced and innovative technique of immobilizing laccase on the surface of the poly-3 hydroxybutyrate and nanofibers and its effect on the dye degradation were observed by Gil et al. (2018) and Wang et al. (2020). The molecular structure of laccases has the presence of four copper atoms, which are differentiated according to their properties in the catalytic mechanism into three types: copper I, copper II, and copper III.

#### Lignin Peroxidase

It falls under the class of oxidative enzymes and can act on the peroxidase enzyme as an electron acceptor. This enzyme is also known as 1,2-bis(3,4-dimethoxyphenyl)propane-1,3-diol hydrogen peroxide. Several researchers have reported the effect of the peroxidase enzyme on dye decolorization and degradation (Kalyani et al., 2011; Falade et al., 2019). LiP can decolorize and mineralize the aromatic compounds, three polychlorinated biphenyls, three- and four-ring polyaromatic hydrocarbons, and most artificial dyes. Lignin peroxidase is the first enzyme to be discovered based on H_2_O_2_ dependence and by the Cα-Cβ cleavage of the compounds. *In vitro* depolymerization of methylated lignin has also been shown by lignin peroxidase. A peroxidase-like activity was seen recently where this enzyme was immobilized on Fe-loaded MOF to decolorize methyl orange, and methylene blue was seen recently (Zhang et al., 2019). This enzyme leads to the oxidation of azo bonds by the oxidation of phenolic groups that produce azo linkages with carbon. This phenolic carbon is then attacked by water that cleaves the molecule and generates phenyldiazene, which can further be oxidized by one electron reaction generating nitrogen.

#### Tyrosinase

This type of enzyme is also called polyphenol oxidase. It is a tetramer structure that consists of four copper molecules and has the binding sites for the two oxygen and aromatic compounds. It was seen that this enzyme helps remove the phenol from the aqueous environment (Roy et al., 2014). The use of tyrosinase in dye decolorization has been observed by various bacteria (Pradhan and Sarkar, 2017). The mechanism of this enzyme occurs in two steps: (1) the first step involves the monophenol hydroxylation, which leads to the formation of o-diphenols called mycophenolate, and (2) the second step is the oxidation of o-diphenols to o-quinone, which is called o-diphenolase. In this mechanism, o-quinone acts as a product and oxygen acts as an oxidant, which can inactivate the activity of tyrosine.

## *In Silico* Studies of Interactions of Dyes With Bacterial Enzymes

*In silico* studies of bacterial enzymes were started from 3D structure generation of enzymes by homology modeling methods and identification of dye structure from different databases. Along with this, a significant step was taken to explore the active or catalytic site of the enzyme, and where exactly the dyes interact. Cyanobacterial azoreductase enzymes of *Nostoc* species were explored computationally, and physicochemical, evolutionary, and structural properties were explored using various bioinformatics tools. An integrated approach of wet and dry laboratories involving computational biology in removing dye is explained in Figure 2. Based on multiple sequences analysis, conserved regions were observed, considered an active site for interactions with dyes. These reasons are from 1 to 11, 40–57, 82–120, and 161–177 amino acid residues of azoreductase (Devi and Adhikari, 2012). It is very important to know the exact mechanism of dyes’ interaction with bacterial enzymes, and *in silico* docking studies are beneficial to understand the mechanism behind it. A recent study of six toxic dyes were shown to degrade by azoreductase and laccase of *Aeromonas hydrophila* and *Lysinibacillus sphaericus* through *in silico* docking tool BioSolveIT-FlexX (Srinivasan et al., 2019). Molecular docking has several applications and significance; it can explain a protein (bacterial enzymes) and ligand (azo dyes) interaction at the computational level (Sridhar and Chandra, 2014). Amino acid residues of enzymes interacting with dyes can be easily predicted by docking. Computational identification of catalytic site in FMN-dependent NADH azoreductase of *E. coli* and its interaction with azo dyes proves that Phe-172, Glu-174, Lys-145 and Lys-169, and Asp-146 amino acids of enzymes were involved in the interaction with azo dyes at the cellular level (Adhikari et al., 2014). A comparative study of six different dyes of industrial origin with laccase enzymes of marine and terrestrial origin was performed, and interaction at the molecular level was explored (Castaño et al., 2016). Similarly, molecular docking between laccase and azoreductase of *Aeromonas hydrophila* and *Lysinibacillus sphaericus* was performed, and atomic level interaction was explored using computational tools (Srinivasan and Sadasivam, 2018). *In silico* approaches of enzymes and dye study are vital methods that give a clear idea about interactions at the atomic level. Figure 2 explains the basic steps of docking and the need for computational structures of enzymes and dyes.

## Ecological Importance of Detoxification of Azo Dyes by Microorganisms

The ecological parameters of detoxification of dye include (1) different levels of assessments, i.e., at molecular, organismic, cellular, chronic, and phytotoxicity level; (2) multispecies/single pure species approach; (3) proteomics and genetic level approach; and (4) dye degraded metabolite identification and its characterization. However, the toxicity of azo dye metabolites is different at the different organizational level. For example, dispersive orange one dye showed cytotoxicity and genotoxicity but did not show any toxicity at the organism level (Ferraz et al., 2011).

### Cellular and Molecular Toxicity

Along with applications, the dyes cause various hazardous and harmful effects. Various molecular assays were used to determine the mutagenicity of the metabolites formed at the molecular level, like Ames test, TUNEL assay, and *Allium cepa* assay. The mechanism of toxicity at different levels is explained in Figure 2. Mostly molecular assay provides very little information on ecological importance but does provide the molecular result at the population level. It was reported that Azure B dye had shown its toxicity in the biological cells by the intercalation between the DNA and the membrane lipid (Haq and Raj, 2018). Various toxic substances are present in the dyes, which have caused bleeding, nausea, and ulceration and have affected human health (Zhuang et al., 2020). Reactive dyes also cause various allergic reactions and toxicity to aquatic life if released in the water, and are mutagenic and carcinogenic toward humans (Das and Mishra, 2017). Azo dyes, being the largest groups of dyes, are the most hazardous and toxic. These azo dyes have many ill effects on humans like cancer, chromosomal aberrations in the cells, splenic carcinomas, irritation of eyes, hepatocarcinomas, and nuclear abnormalities in experimental animals, and also these types are highly soluble in water, which can reach humans through the food chain and can cause fever, renal damage, and cramps (Sudha et al., 2014). Some of the dispersive dyes, like Dispersive blue 291, cause genotypic effects and cause fragmentation of DNA in human cells and form the micronuclei, and Disperse red 1 and Disperse orange 1 increase the micronuclei frequencies in human lymphocytes (Sudha et al., 2014). Moreover, the molecular studies performed for the natural detoxification of azo dyes give insight into a better understanding of the environmental processes that aim at reliable industrialization. Therefore, the facts previously mentioned state that chemical structure and toxicity molecular assay might help recognize the ecologic processes that play a significant role in the fate of the dye. Cellular toxicity of the metabolites produced by the microorganisms mainly depends on the species strain and the environment of degradation. This type of toxicity mainly occurs in plants and animals. For example, reactive dyes affect humans and also the photosynthetic activity of aquatic plants because these reactive dyes contain metals and chlorides (Hossen et al., 2019). Similarly, it has been reported if the triphenylmethane dyes are inappropriately disposed of in the aqueous ecosystem, it can affect the BOD and COD of the aqueous ecosystem and can also affect flora and fauna, which will indirectly lead to environmental problems (Roy et al., 2018). Major studies on the toxicity related to humans are based on cellular level research. Acid Red 337, an azo dye, is recorded as the most harmful organic matter to the environment by the European Chemical Agency (Ewida et al., 2019). Disperse dyes, if they are released in the water, leads to bioaccumulation (Chittal et al., 2019). For a better understanding of the mechanism followed by organism and plant species, the account of proteomic analysis plays a very important role, as discussed earlier, along with the identification of metabolites.

### Chronic Toxicity and Phytotoxicity

The chronic and acute toxicity assay can also identify the ecotoxicity of the azo metabolite formed. Apart from microbes, various high organisms such as amphibians and fishes are also used as bioindicators to assess the toxicity of dyes. Zhang et al. (2012) showed the acute toxicity induced by dye effluent in zebrafish that highlighted the incompetence of sewage treatment plants in eliminating the toxic compounds from sludge. However, the products formed by the degradation of m-phenylenediamine and p-phenylenediamine showed the toxicity of the specific organ during chronic exposure. Furthermore, various aquatic organisms like *Artemia salina*, *Meloidogyne incognita*, and *Daphnia magna* have been used as model organisms to study the toxic impacts (Ayed et al., 2011; Pathak et al., 2014). Assessment of phytotoxicity has also been useful in determining dye detoxification. Majorly, the metabolite-induced toxicity is skewed for the analysis of phytotoxicity because of the significant differences in the characteristics of physical and chemical properties of the metabolites and dyes. These existing differences limit the advantages of the phytotoxicity analysis and affect the plant uptake of a compound. For example, the metabolites formed that are aromatic are mostly hydrophobic and are least available phytologically as they show high affinity for the organic matter of soil (Briggs, 1981). However, the azo dyes are mostly phytoavailable because they show high hydrophobicity. From these observations, we can conclude that unidentified metabolites and phytotoxicity analysis of dye effluent might not be considered the best detoxification methods by microbes.

## Conclusion and Future Perspective

Accumulated dyestuff and wastewater of dye leads to environmental pollution and are hazardous to humans. Therefore, there is an urgent need for cost-effective remedial methods. Microbial degradation or enzymatic degradation is the most favored method to overcome the accumulation of dye because they are eco-friendly, cheaper, and do not produce excessive sludge. Besides, bacterial degradations are preferred because they are easy to grow and have a high hydraulic retention time. Furthermore, they are efficient in treating wastewater containing excessive organic pollutants. *In silico* docking studies of different enzymes with different dyes can give us a virtual picture of their interactions. Based on this, we can identify the best bacterial strain that can degrade the dye perfectly. With the help of such computational analysis, we can identify the bacterial strains and grow where a specific dye is found. Thus, field experiments will be more focused, and chances of success will be increased. In fields, the bacterial degradation of dyes can occur under three conditions that are aerobic, anaerobic, and the combined method. Each method has its advantages and disadvantages, as discussed. Under anaerobic conditions, the reductive cleavage of azo bonds leads to the formation of toxic aromatic amines, which cannot completely decolorize the dye. In general, anaerobic–aerobic biological strategies are appropriate for the remedy of azo dye–containing wastewaters. Many parameters play their role in decolorization performance, so optimization is necessary to check which parameters are fast and best. Different approaches are now getting more attention, such as nanotechnology and bio-products that can potentially remove toxic dyes from the environment.

Various oxidoreductase enzymes play their role in degradation. Moreover, the strategies of molecular biology and biochemistry along with genomics, proteomics, and bioinformatics can be coupled, enhancing decolorization efficiency. Many researchers are working on a new innovative method for the biodegradation and detoxification of dye so that it can have the least impact on the ecology, environment, and humans. Current studies have clearly stated that the azo dyes do not have a fixed ecological fate. It is mostly affected by environmental factors, proteins/genes, chemical structure, and microbial actions. Various dye effluents that get released into the environment have harmful effects. Therefore, researchers need to assess the intermediate aromatic metabolites produced at the cellular, molecular, and organism toxicity levels to study their ecological impact. Furthermore, there is no universal solution to the remediation problem until and unless the industries themselves emphasize developing the required biological treatments for detoxification. If the researchers and the processing plants undertake the appropriate strategies, this will lead to the solution and control of the industrial effluents released into the environment.

## Author Contributions

AMi and AV were involved in conceptualization, investigation, and validation. AMi, ST, AS, and NJ were involved in resources, writing—original draft, writing—review and editing, visualization, project administration, and supervision. SS, AMa, and KS were a part of writing—original draft, writing—review and editing, and resources. AMi and NJ were involved in writing—original draft and editing. All authors read and approved the final review article.

## Conflict of Interest

AMa was employed by the company Prathista Industries Limited. The remaining authors declare that the research was conducted in the absence of any commercial or financial relationships that could be construed as a potential conflict of interest.

## Publisher’s Note

All claims expressed in this article are solely those of the authors and do not necessarily represent those of their affiliated organizations, or those of the publisher, the editors and the reviewers. Any product that may be evaluated in this article, or claim that may be made by its manufacturer, is not guaranteed or endorsed by the publisher.
